# Staged Surgical and Endovascular Management of Parkes–Weber Syndrome to Preserve Limb Function: A Case Report

**DOI:** 10.70352/scrj.cr.26-0049

**Published:** 2026-06-03

**Authors:** Kyokei Fuchizawa, Shinsuke Kikuchi, Takayuki Uramoto, Kazuki Takahashi, Hiroya Moriyama, Shunta Ishitoya, Yuri Yoshida, Hisashi Uchida, Nobuyoshi Azuma

**Affiliations:** 1 Department of Vascular Surgery, Asahikawa Medical University, Asahikawa, Hokkaido, Japan; 2 Department of Radiology, Asahikawa Medical University, Asahikawa, Hokkaido, Japan; 3 Department of Cardiovascular Surgery, Sapporo Kosei Hospital, Sapporo, Hokkaido, Japan

**Keywords:** Parkes–Weber Syndrome, Klippel–Trenaunay–Weber Syndrome, arteriovenous malformations, Klippel–Trénaunay Syndrome

## Abstract

**INTRODUCTION:**

Parkes–Weber Syndrome (PWS) is a rare congenital vascular disorder of unknown etiology for which no established curative treatments currently exist. High-flow arteriovenous malformations (AVMs) in PWS can lead to severe complications, including the need for major limb amputation.

**CASE PRESENTATION:**

A woman in her 50s presented with progressive swelling, severe pain, and impaired ambulation in her right thigh. She was diagnosed at age 14 with varicose veins in her right lower limb and a 1.5-cm limb-length discrepancy, with the right leg longer than the left, and later developed an arteriovenous fistula at age 23. By age 54, examination revealed extensive swelling and hardening of the thigh, with CT imaging showing numerous abnormal vessels forming a nidus and a ruptured hematoma measuring 17 × 14 × 18 cm. She was referred to our facility after unsuccessful attempts to remove hematoma removal at a previous institution and was diagnosed with PWS. Preoperative embolization of multiple niduses was performed to reduce blood flow, although perfusion to other niduses persisted. Hematoma removal was conducted in 2 stages. The first surgery involved securing arterial flow with a stent graft and partial excision of the hematoma (13 h 39 min; blood loss: 9105 mL). Nineteen days later, a second surgery was performed to remove approximately 90% of the hematoma while preserving the remaining wall to avoid complications (8 h 14 min; blood loss: 5533 mL). The patient recovered without complications and was discharged 2 weeks following the second surgery. Minor delays in wound healing resolved within 6 months, and she remains recurrence-free at 48 months.

**CONCLUSIONS:**

This case highlights the complexity of managing PWS and underscores the importance of individualized, multidisciplinary care.

## Abbreviations

AVMs
: arteriovenous malformations
CMs
: capillary malformations
ISSVA
: The International Society for the Study of Vascular Anomalies
KTWS
: Klippel–Trénaunay–Weber Syndrome
KTS
: Klippel–Trénaunay Syndrome
PWS
: Parkes–Weber Syndrome
SFA
: superficial femoral artery

## INTRODUCTION

KTWS is a congenital vascular disorder classically characterized by unilateral limb hypertrophy, port-wine stains, and varicose veins.^1)^ Recent advancements in the classification of vascular anomalies have redefined KTWS by distinguishing it a separate conditions based on the flow dynamics of vascular malformations. KTS involves low-flow malformations of veins, capillaries, and lymphatics, while PWS is characterized by high-flow AVMs. These high-flow AVMs in PWS can lead to severe complications, including refractory venous ulcers, high-output heart failure, and a significantly elevated risk of limb amputation. In contrast, KTS generally carries a lower risk of such complications. Accurately differentiating between these conditions is essential for proper diagnosis and treatment. This paper focuses on a case of PWS presenting with severe lower limb complications and details the surgical and endovascular interventions performed, as well as a review of the relevant literature.

## CASE PRESENTATION

**Fig. 1:**
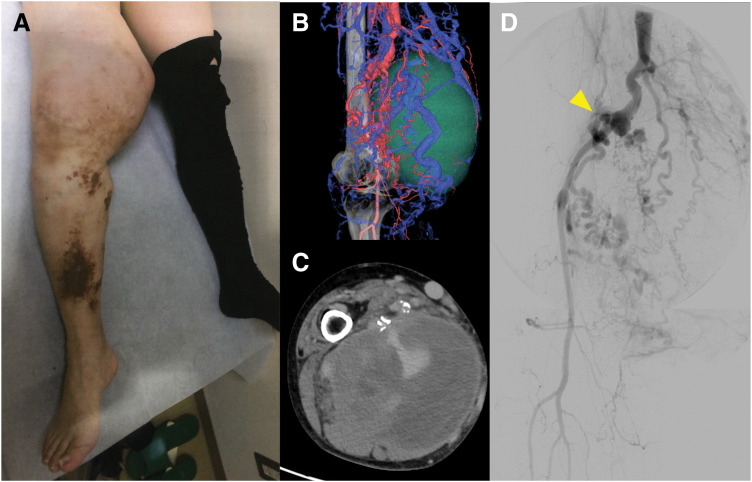
Preoperative findings. Her right thigh was markedly swollen, and the lower limb showed port-wine stains, varicose veins, and overgrowth (**A**). Contrast-enhanced CT imaging demonstrated multiple abnormal arteries and veins forming AVMs (**B**). The mass of her right thigh was identified as a hematoma resulting from the rupture of AVMs (**C**). DSA revealed tortuous abnormal vessels (yellow arrowhead) originating from the SFA and the popliteal artery, forming a nidus of vascular malformations, and also demonstrated aneurysmal formation (**D**). AVM, arteriovenous malformation; SFA, superficial femoral artery

A 54-year-old woman was referred to our hospital due to progressive swelling and pain in her right thigh, which caused significant difficulty in walking. In her teens, she was treated for varicose veins in her right lower limb and was noted to have a 1.5-cm limb-length discrepancy, with the right leg longer than the left. Her family history was unremarkable. In her 20s, she underwent coil embolization for an arteriovenous fistula in her right lower limb. Beginning in her 50s, her right thigh began to swell progressively. The increasing size of the mass led to severe pain and impaired ambulation, prompting her to visit her previous physician (**Fig. 1A**). The mass was identified as a hematoma resulting from the rupture of AVMs. Although an attempt was made to remove the hematoma at the previous institution, the procedure was aborted due to uncontrollable bleeding. She was subsequently referred to our hospital for the removal of a hematoma. Notably, she had not been diagnosed with PWS until this point.

**Fig. 2:**
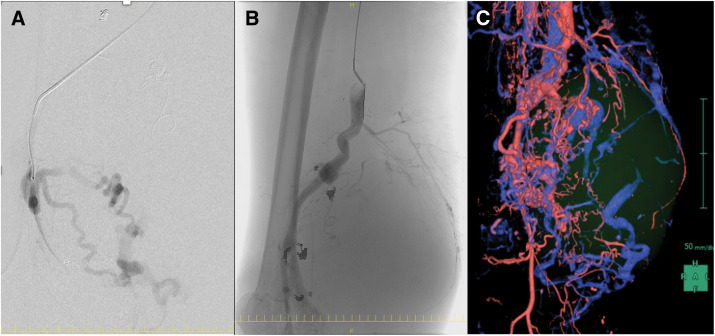
Coil embolization findings. A total of 29 coils were used to embolize abnormal vessels extending from the SFA to the hematoma over 8 h (**A**). Post-embolization DSA showed a reduction in the AVM extending the hematoma (**B**). Unfortunately, post-embolization CTA revealed the persistence of numerous arteriovenous fistulas (**C**). AVM, arteriovenous malformation; SFA, superficial femoral artery

The physical examination revealed port-wine stains, overgrowth of the right lower limb, and varicose veins, alongside marked hypertrophy of the right thigh. Notably, multiple small CMs were not observed; the patient exhibited only a solitary port-wine stain on the affected limb. Contrast-enhanced CT imaging revealed multiple abnormal arteries and veins forming AVMs, including aneurysm formation in the distal thigh (**Fig. 1B** and **1C**). A large hematoma, measuring 17 × 14 × 18 cm, was observed with evidence of contrast enhancement within the mass. DSA further revealed tortuous abnormal vessels originating from the SFA and popliteal artery, forming a nidus of vascular malformations (**Fig. 1D**). The diagnosis of PWS was confirmed based on the presence of arteriovenous fistulas, varicose veins, and overgrowth of the right lower limb. The enlarging hematoma posed a significant risk of major limb amputation, including transfemoral or hip disarticulation. Therefore, a treatment plan was developed to control the arteriovenous fistulas, achieve maximum excision, and remove the massive hematoma. Anticipating massive intraoperative bleeding, the initial step involved coil embolization of the nidus using interventional radiology to control blood flow. Abnormal vessels extending from the SFA to the hematoma were embolized using a total of 29 coils over 8 h (**Fig. 2A** and **2B**). However, post-embolization CTA revealed the persistence of numerous arteriovenous fistulas (**Fig. 2C**). Subsequently, open surgery was planned to resect the nidus and excise the hematoma. Preparations included arrangements for extensive blood transfusions and the potential deployment of stent grafts to address the dilated femoral and popliteal arteries (Excluder contralateral legs, Gore PLC121400J, approved by the Clinical Ethics Committee of Asahikawa Medical University, #20005).

**Fig. 3:**
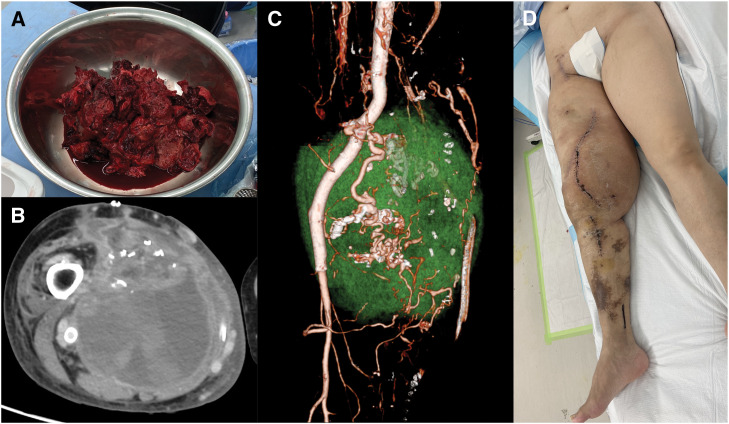
Removed hematoma and the first postoperative findings. The large hematoma was incised, and its contents were removed (**A**). Postoperative CTA revealed residual hematoma in the distal thigh, raising concerns about potential re-expansion in the future (**B**). Given the arterial seal achieved with the stent grafts, the remaining arteries were presumed to be osseous origin (**C**). While the swelling in the thigh improved compared to preoperative levels, the improvement in QOL regarding aesthetic outcomes was limited (**D**).

Under general anesthesia, surgery was initiated (**Video 1**). The common femoral artery and the popliteal artery and vein below the knee were secured. The hematoma was dissected, with concurrent ligation of abnormal vessels. During dissection, an aneurysm was inadvertently damaged, leading to active bleeding. To manage this, stent grafts were deployed to seal the abnormal vessels. Two 10-mm diameter, 10-cm-long VIABAHN stent grafts (Gore JHHR101002J) were inserted peripherally, and a reversed Gore contralateral limb stent (proximal diameter 16 mm, distal diameter 14 mm) was placed to address the dilated SFA and aneurysm. After bleeding was controlled, the hematoma was incised, and its contents were removed (**Fig. 3A**). Arterial bleeding from the medial side of the hematoma was sutured for hemostasis. Owing to the thick and fibrotic nature of the hematoma wall, complete excision was not feasible in a single session, and a staged approach was adopted (operation time: 13 h 39 min; blood loss: 9105 mL). A large hematoma was successfully excised, and the pathological findings were consistent with the diagnosis of a hematoma. Postoperative CTA revealed a residual hematoma in the distal thigh, raising concerns about potential re-expansion in the future (**Fig. 3B**). Given the arterial seal achieved with the stent grafts, the remaining arteries were presumed to be of osseous origin (**Fig. 3C**). Although the diameter of the affected limb improved postoperatively, the residual hematoma remained, resulting in limited improvement in QOL, particularly in terms of aesthetic outcomes (**Fig. 3D**). The second surgery, performed 19 days later, focused on removing the remaining hematoma wall (**Video 2**). Although a portion of the posterior wall was adherent to surrounding muscles and nerves, approximately 90% of the hematoma wall was successfully excised. A small portion was left intact to avoid complications (operation time: 8 h 14 min; blood loss: 5500 mL).

**Fig. 4:**
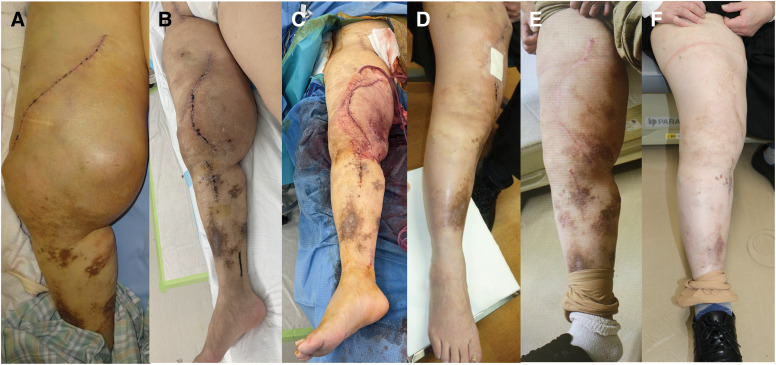
The thigh diameter improvement findings. Preoperative (**A**). After the first surgery (**B**). Immediately after the second surgery (**C**). One month after the second surgery, a minor wound healing delay was observed (**D**). Complete wound healing was achieved 6 months after the second surgery (**E**). At 30 months after the second surgery, the patient remained recurrence-free without the need for further intervention (**F**).

Following stent graft placement in the SFA, the patient was maintained on long-term single antiplatelet therapy with aspirin 100 mg once daily, and no bleeding complications occurred during the 48-month follow-up period despite residual AVMs. The postoperative course was uneventful. The diameter of the thigh decreased markedly after each staged procedure, and she regained independent ambulation, allowing discharge 2 weeks after the second surgery. Minor delays in wound healing resolved completely within 6 months. Although postoperative sensory numbness persisted in the distribution of the left saphenous nerve, making precise pain assessment difficult, she reported no activity-limiting discomfort. She returned to work 1 month after surgery and resumed bicycling after 3 months. Mild residual edema persisted, for which she consistently wore thigh-high compression stockings. At 48-month follow-up, she remained recurrence-free with no complications (**Fig. 4**). Importantly, the staged intervention avoided major limb amputation and resulted in substantial improvements in functional status, cosmetic appearance, and overall QOL.

## DISCUSSION

KTS and PWS are distinct congenital vascular anomalies; however, their overlapping phenotypic features, namely limb hypertrophy, port-wine stains, and varicosities, frequently engender diagnostic ambiguity.^2)^ The ISSVA classification provides a critical framework for differentiation by categorizing vascular malformations based on flow dynamics: low-flow lesions, including capillary, venous, and lymphatic malformations, typify KTS, whereas PWS is distinguished by high-flow AVMs.^3,4)^ The presence of high-flow AVMs in PWS carries profound hemodynamic consequences, predisposing patients to high-output cardiac failure, ischemic pain, refractory ulceration, and, in severe cases, major limb amputation.^4)^ The present case did not show any clinical or imaging evidence of cardiac overload. Both chest radiography and transthoracic echocardiography showed normal findings, including a normal cardiothoracic ratio and cardiac output. Therefore, high-output heart failure was not observed in this case. Indeed, reported amputation rates in PWS range from 10% to 16.7%,^5)^ underscoring the importance of early recognition and proactive intervention. In the present case, the patient’s progressive symptoms and massive hematoma necessitated a staged therapeutic approach, combining preoperative embolization with surgical resection to achieve limb preservation and symptomatic relief.

An accurate diagnosis of PWS requires comprehensive clinical and radiologic evaluation. Although both syndromes may exhibit similar cutaneous and structural manifestations, PWS is uniquely characterized by high-flow indicators such as pulsatile varices, palpable thrills, and localized hyperthermia.^6,7)^ Advanced imaging modalities, including contrast-enhanced CT and MRI, are instrumental in delineating lesion flow characteristics, while catheter-based angiography remains the gold standard for mapping vascular architecture and guiding interventional strategies.^6–8)^

Therapeutic objectives in PWS focus on optimizing QOL and mitigating complications. A multidisciplinary regimen integrating endovascular embolization with surgical excision yields favorable outcomes, particularly when addressing high-flow AVMs.^9,10)^ In this case, extensive coil embolization was performed to reduce intraoperative hemorrhage, followed by the strategic deployment of stent grafts to occlude aberrant arterial inflow. Although contemporary literature indicates that n-butyl-2-cyanoacrylate (nBCA) is used in approximately 69% of PWS embolization procedures and that combined approaches using ethanol or nBCA with coils have shown favorable outcomes,^10,11)^ we selected coils as the primary embolic agent in this case for several reasons. First, definitive surgical interruption of the AVMs was planned; therefore, the preoperative goal of embolization was not complete nidus eradication but rather a reduction of arterial inflow to the hematoma to facilitate a safer operation. Second, the patient had markedly dilated and tortuous venous channels surrounding the hematoma, raising concern that liquid embolic agents such as nBCA or ethanol could not be selectively delivered and might migrate into the abnormal venous system. This posed a substantial risk of non-target embolization. In contrast, coils allowed controlled occlusion of major feeding arteries while minimizing the risk of unintended venous embolization. Nevertheless, it is possible that the use of liquid embolic agents or combined techniques might have further reduced intraoperative bleeding, which remains an important consideration for future cases. Although stent grafts provide effective hemostasis, their use necessitates antiplatelet therapy and carries inherent risks, warranting judicious patient selection.^9)^

Notably, arterial aneurysms are observed in approximately 10% of PWS cases, with stent graft placement reported in only a minority (4%)^9)^ [8]. Furthermore, AVMs originating from osseous structures are refractory to conventional tourniquet control, necessitating meticulous preoperative planning and intraoperative vigilance. Emerging genetic insights have further elucidated the pathophysiology of PWS, with loss-of-function mutations in the RASA1 gene, which encodes the p120-Ras GTPase-activating protein, implicated in its etiology.^5,12)^ These mutations may be inherited in an autosomal dominant fashion or arise as postzygotic mosaic variants. In this patient, the cutaneous lesion visible on the lower limb (**Fig. 4A**) represented a broad, solitary port-wine stain rather than multiple small CMs. Multiple CMs, which are characteristic of RASA1-related CM–AVM syndrome, were not observed.^13)^ Although genetic testing for RASA1 mutations was not performed, the absence of multiple CMs suggests a low likelihood of an underlying RASA1 variant. This remains a limitation, but genetic evaluation may still be considered for counseling purposes in selected cases. Next-generation sequencing facilitates precise molecular diagnosis and may inform future therapeutic innovations.^14)^

This case exemplifies the formidable challenges inherent in managing PWS, particularly in the context of extensive AVMs and hemorrhagic complications. It reinforces the necessity of individualized treatment paradigms, underpinned by multidisciplinary collaboration and advanced surgical planning, to achieve durable clinical improvement and long-term limb preservation.

## CONCLUSIONS

PWS is an uncommon congenital vascular anomaly characterized by high-flow AVMs. The presence of high-flow AVM leads to various complications and is associated with a high rate of limb amputation. The standard treatment for PWS has not been established, and the primary treatment goals are to improve QOL and reduce complications. Surgical resection of AVMs is useful for clinical improvement but carries a risk of massive intraoperative bleeding. Therefore, the combination of endovascular embolization and surgical resection is effective. Treatment should be individualized for each patient, and a comprehensive approach involving multidisciplinary collaboration is essential.

## Supplementary Materials

### Supplementary Video

Video 1. Intraoperative video of the first-stage surgery showing deployment of stent grafts to control arterial bleeding from high-flow arteriovenous malformations and partial evacuation of the hematoma.

Video 2. Intraoperative video of the second-stage surgery demonstrating excision of the residual hematoma wall and achievement of definitive hemostasis.

## DECLARATIONS

### Availability of data and materials

The data used for this case report are available from the corresponding author upon request.
